# Comparative genomics and transcriptomics insight into myxobacterial metabolism potentials and multiple predatory strategies

**DOI:** 10.3389/fmicb.2023.1146523

**Published:** 2023-05-05

**Authors:** Chunling Wang, Yi Xiao, Yong Wang, Yumin Liu, Qing Yao, Honghui Zhu

**Affiliations:** ^1^College of Life Science, Huizhou University, Huizhou, Guangdong, China; ^2^Key Laboratory of Agricultural Microbiomics and Precision Application (MARA), Guangdong Provincial Key Laboratory of Microbial Culture Collection and Application, Key Laboratory of Agricultural Microbiome (MARA), State Key Laboratory of Applied Microbiology Southern China, Institute of Microbiology, Guangdong Academy of Sciences, Guangzhou, Guangdong, China; ^3^College of Horticulture, South China Agricultural University, Guangdong Province Key Laboratory of Microbial Signals and Disease Control, Guangzhou, Guangdong, China

**Keywords:** myxobacteria, predation, protein secretion systems, Tad pilus, secondary metabolites, genomics and transcriptomics

## Abstract

Myxobacteria are part of the phylum Myxococcota, encompassing four orders. Most of them display complex lifestyles and broad predation profiles. However, metabolic potential and predation mechanisms of different myxobacteria remains poorly understood. Herein, we used comparative genomics and transcriptomics to analyze metabolic potentials and differentially expressed gene (DEG) profiles of *Myxococcus xanthus* monoculture (Mx) compared to coculture with *Escherichia coli* (MxE) and *Micrococcus luteus* (MxM) prey. The results showed that myxobacteria had conspicuous metabolic deficiencies, various protein secretion systems (PSSs) and the common type II secretion system (T2SS). RNA-seq data demonstrated that *M. xanthus* overexpressed the potential predation DEGs, particularly those encoding T2SS, the tight adherence (Tad) pilus, different secondary metabolites (myxochelin A/B, myxoprincomide, myxovirescin A1, geosmin and myxalamide), glycosyl transferases and peptidase during predation. Furthermore, the myxalamide biosynthesis gene clusters, two hypothetical gene clusters and one arginine biosynthesis clusters were highly differential expressed in MxE versus MxM. Additionally, homologue proteins of the Tad (kil) system and five secondary metabolites were in different obligate or facultative predators. Finally, we provided a working model for exhibiting multiple predatory strategies when *M. xanthus* prey on *M. luteus* and *E. coli*. These results might spur application-oriented research on the development of novel antibacterial strategies.

## Introduction

Myxobacteria are fascinating Gram-negative bacteria, and are distributed all over the world, such as terrestrial, freshwater and marine habitats, etc., (Albataineh and Stevens, 2018; Mohr, 2018). They are well known for their complex social behaviors, including (but certainly not limited to) multicellular fruiting body formation, motility and predation (Muñoz-Dorado et al., 2016). Myxobacteria have two complementary flagella-independent motility forms, adventurous motility, which drives the movement of individual cells, and the type IV pili (T4P)-dependent motility, which drives the movement of large cell groups (Muñoz-Dorado et al., 2016). Myxobacteria have excellent production abilities of novel bioactive secondary metabolites and enzymes (Wrotniak-Drzewiecka et al., 2016; Moghaddam et al., 2018). Most myxobacterial genome sizes range from 9.0 to 16.0 Mbp with genomic DNA G + C contents ~70.0 mol% (Garcia et al., 2014; Sharma et al., 2016; Wang et al., 2022). Recently, myxobacteria are reclassified as the phylum Myxococcota, comprising the orders Myxococcales, Polyangiales, Nannocystales and Haliangiales (Waite et al., 2020). Due to the difficulties in isolation, only seven families and 19 genera have been validly published in the LPSN database.^1^ According to their nutritional behaviors and specialization in the degradation of biomacromolecules, members of the phylum Myxococcota are divided into two groups: proteolytic and cellulolytic myxobacteria (Reichenbach et al., 2006; Mohr et al., 2018).

Proteolytic myxobacteria are generalists that are able to feed on a broad range of bacteria, yeasts and filamentous fungi (Thiery and Kaimer, 2020). They employ a multilayered predatory strategy to kill and lyse prey (Pan et al., 2013; Arend et al., 2020; Zhang et al., 2020). Antibiotics, bacteriolytic enzymes and hypothetical proteins delivered by outer membrane vesicles (OMVs) play important roles (Thiery and Kaimer, 2020). These metabolites (e.g., myxovirescin, myxoprincomide and corallopyronin) and lyases (*β*-1,6-glucanase GluM and a family 19 glycoside hydrolase llpM) have been directly implicated to be involved for killing and degrading prey (Xiao et al., 2011; Müller et al., 2016; Li et al., 2019; Arend et al., 2020). Furthermore, the prey cell wall and protein production are primary targets of the *Myxococcus xanthus*’s attack (Livingstone et al., 2018). In addition, a recent survey combined action of the tight adherence (Tad)-like and a type 3-like secretion system (T3SS*) induces prey cell death and lysis (Seef et al., 2021; Thiery et al., 2022). Based on the broad spectrum of prey that can be utilized by myxobacteria, different molecular mechanism, acting either in isolation or synergistically, are required to prey on different species.

Considering that myxobacterial predation involve contact-dependent killing before degrading, we hypothesize that the key genes associated with contact-dependent killing could be determined by core gene families, while genes that affect the predation efficiency of different groups could be caused by accessory gene families. Additionally, myxobacteria harbor high amounts of uncultivated groups, which are difficult to study due to a lack of appropriate culture conditions. In order to gain a deeper understanding of myxobacterial characteristics and predation mechanisms, herein, we utilize a combination of comparative genomics and transcriptomics to analyzemetabolite potentials and differentially expressed gene (DEG) profiles of *M. xanthus* monoculture (Mx) compared to coculture with *Escherichia coli* (MxE) and *Micrococcus luteus* (MxM) prey. We further compared the predatory and non-predatory bacterial genomes for the presence of the potential predatory effectors. Finally, we provide a work model for exhibiting different predatory strategies of *M. luteus* and *E. coli* by *M. xanthus*.

## Materials and methods

### Genome annotation and metabolic reconstruction of the phylum Myxococcota

To explore myxobacterial metabolic potentials, we selected and downloaded 17 genomes including 9 of Myxococcales, 4 of Polyangiales, 3 of Nannocystales and 1 of Haliangiales within the Myxococcota from NCBI database.^2^ To keep uniformity in the analysis, proteins encoded in all genomes were predicted using Prokka (v1.13) with default parameters. Transfer RNA-coding regions were designated using tRNAscan-SE 2.0 sever (Lowe and Eddy, 1997). The genome-based metabolic potentials and different protein secretion systems (PSSs) including the type I, II, III, IV and VI secretion systems (T1SS, T2SS, T3SS, T4SS, and T6SS) were reconstructed by uploading protein files to the Kyoto Encyclopedia of Genes and Genomes (KEGG) sever using BlastKOALA^3^ to retrieve KEGG ortholog (KO) numbers. Genes related to the type IV filament (TFF) superfamily including T2SS, T4P, the Tad (kil) pilus and the mannose-sensitive hemagglutinin pilus (MSH) were detected using MacSyFinder v1.0.2 program (Torres et al., 2014). A full description of the MacSyFinder program and the models can be found in.^4^

### Predation of cells for transcriptomic experiments

To explore the mechanisms by which myxobacteria prey on different bacteria, we selected a model strain *M. xanthus* DK1622, the Gram-negative bacteria *E. coli* ATCC 8739 and Gram-positive bacteria *M. luteus* NCTC2665 as representative strains for predatory assays. *M. xanthus* was grown in CTT medium (casitone 10 g/L, MgSO_4_^.^7H_2_O 8 mM, potassium phosphate buffer (pH 7.6) 1 mM, tris–HCl (pH 7.6) 10 mM) at 30°C and 200 rpm for 5 days. *E. coli* and *M. luteus* were cultivated in NA medium (beef extract 3.0 g/L, peptone 5.0 g/L, glucose 2.5 g/L, pH 7.2) at 30°C and 200 rpm for 2 days. Cells were sedimented via centrifuged (6,000 rpm for 8 min), washed, and concentrated in TPM buffer (tris–HCl (pH 7.6) 10 mM, K_2_HPO_4_ 1 mM and MgSO_4_ 8 mM) to a final concentration of 1 × 10^9^ cells/mL for predators and 5 × 10^10^ cells/mL for prey. *Myxococcus xanthus* suspensions (final concentration of 1 × 10^8^ cells/mL) and the prey suspensions (final concentration of 4.5 × 10^10^ cells/mL) were thoroughly mixed and spotted on different WAX agar plates (CaCl_2_·2H_2_O 1 g/L, HEPES 20 mM and agar 15 g/L, pH 7.2) incubated at 30°C for 4, 9 and 12 h, respectively. The mixed cultures were observed by light microscope (DM6/MC190, Leica). Meanwhile, *M. xanthus* suspensions (final concentration of 1 × 10^9^ cells/mL) were also spotted on WAX agar plates incubated at 30°C. According to microscopic observations, the monoculture and coculture cells incubated for 12 h were then harvested and stored at −80°C for further transcriptomic analysis. Each sample was collected in triplicate (*n* = 3).

### RNA extraction and transcriptomic sequencing

RNA was extracted using an miRNEasy mini kit (Qiagen 217,004), rRNA was removed using a Ribo-Zero magnetic kit from Epicentre (MRZB12424), and cDNA library construction was performed with a TruSeq Stranded mRNA library preparation kit from Illumina (RS-122-2,101). Sequencing was carried out on a HiSeq sequencer at Novogene Co., Ltd. (Beijing, China). The raw reads were uploaded in the NCBI Sequence Read Archive (SRA) database under BioProject accession numbers SRR19236891-SRR19236899.

### Transcriptome mapping and differential expression analysis

Trimmomatic was used to clean the raw data before downstream processing to remove adaptor sequences and trailing based with quality thresholds below 20, perform sliding window trimming (with parameters 4:15), and remove reads less than 36 bp in length. After cleaning, the remaining paired reads were mapped to the complete genome of *M. xanthus* DK1622 (CP000113) using Bowtie2 software reported by Langmead et al. (2019) to calculate expression values. The output fragments per kilobase of transcript per million mapped reads (FPKM) were calculated for further analysis. Differences in relative gene expression were assessed, significantly upregulated and downregulated genes were defined using a false discovery rate of less than 0.001, a *p* value of multiple hypothesis test (padj) < 0.05 & |log2(fold)-change| > 0. Carbohydrate-active enzymes were identified by dbCAN database using HMMER3 annotation (E-value<1e-15, coverage>0.35). Secondary metabolite gene clusters were predicted by antiSMASH 5.0 websever. The other related genes were from references (Konovalova et al., 2010; Muñoz-Dorado et al., 2019).

### Comparative genomic analyses

To explore the predatory and non-predatory bacteria for the presence of the potential predatory effectors, all 107 genomes including 92 from the four orders within the Myxococcota, ten from other obligate and facultative predatory bacteria including Lysobacterales, Bradymonadales, Herpetosiphonales and Bdellovibrionales, and five from non-predators obtained using the same isolation method with myxobacteria, including *Chitinophaga* (Lv et al., 2018, 2019), *Deminuibacter* (Wang et al., 2019) and *Chryseolinea* (Wang et al., 2018), were downloaded and selected from the NCBI database. These genomes were subjected to annotation using eggNOG-mapper (v2) with “diamond blastp” against eggNOG database at default settings. Homologue proteins were determined by OrthoFinder (v2.5.2) based on the “Diamond blastp” (−id 50) and “MCL” (-I 1.5) algorithms. The Tad (kil) genes of different species were found and extracted using MacSyFinder v1.0.2 program. Phylogenomic tree based on 92 bacterial core genes was reconstructed using UBCG pipeline (v3.0) (Na et al., 2018) and further processed in Interactive Tree Of Life (iTOL) (Letunic and Bork, 2016) and illustrator.

### Quantitative real-time PCR

Analysis of the potential predation DEGs was conducted by extracting total RNA from bacteria with HiPure Bacterial RNA Kit (Magen, Guangzhou, China) following the protocol as described by manufacturer. cDNA was synthesis with HiScript 1st Strand cDNA Synthesis Kit (Vazyme Biotech, Co., Ltd.#R111-01, China) after DNaseI treatment to remove genomic DNA contamination. It was then used as a template for qRT-PCR, which was performed with a PrimeScriptTM RT reagent Kit (Perfect Real Time) (Takara, Code No.: RR037A, Japan) on the Applied Bio-systems (QuantStudio 5, United States). The qRT-PCR response procedures adopt a two-step cycling protocol with a procedure of 95°C for 3 min, followed by 40 cycles of 95°C for 10 s and 60°C for 35 s. The data were analyzed by using software QuantStudioTM Design & Analysis Software v1.5.1. The 16S rRNA gene was selected as the internal control. Results obtained from three biological repeats with standard deviation (SD).

## Results and discussion

### The metabolic potentials of the 17 species within the Myxococcota

We analyzed 17 genomes belonging to four orders of the Myxococcota as respective species to explore their metabolism potentials. The genome sizes ranged from 9.1–13.0 Mbp except for three exceptional cases, namely *Simulacricoccus ruber* 17bor14^T^ (6.9 Mbp), *Anaeromyxobacter dehalogenans* 2CP-1^T^ (5.0 Mbp) and *Vulgatibacter incomptus* DSM 22710^T^ (4.4 Mbp) (Supplementary Figure S1). These genomes contained 3,640–9,668 genes, 27–66 peptidases and 30–62 nucleases with genomic DNA G + C contents of 67.4–74.7 mol% (Supplementary Figure S1).

**Figure 1:**
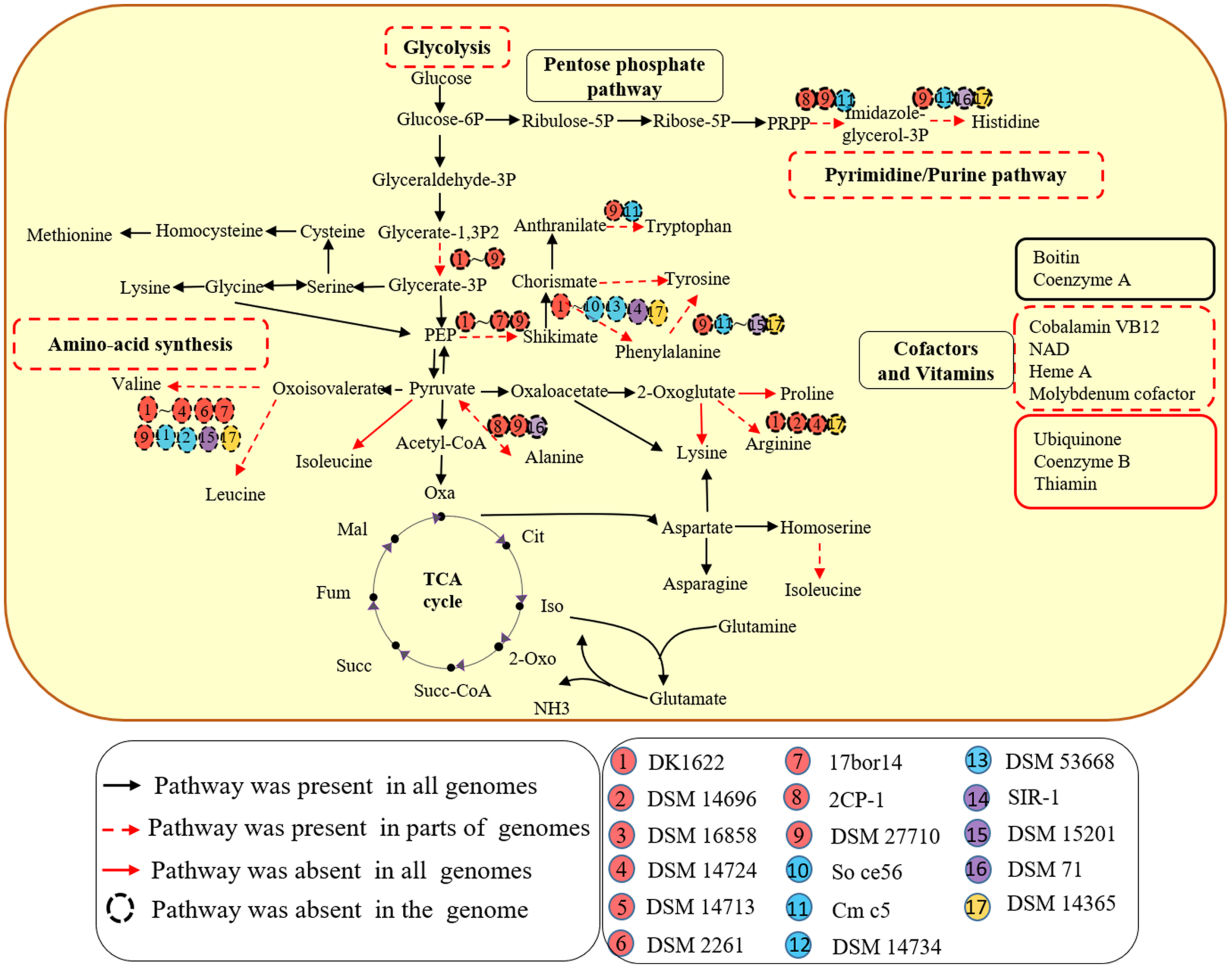
Metabolic potentials of the 17 species of four orders within the Myxococcota. Metabolic prediction was generated by referring to KEGG database interface. Each subgroup of the four orders is depicted as a colored circle. The black arrows indicate the corresponding genes detected for the pathways in all 17 genomes. The red dotted arrows indicate the corresponding genes detected for the pathways in parts of genomes, while the red solid arrows indicate the corresponding genes missing from the pathways in all genomes. The dotted circle indicates the corresponding genes missing from the genome.

All 9 genomes within the order myxococcales lacked the gene for the key enzyme phosphoglycerate kinase (pgk) to synthesize glycerate, which utilizes glycerate-1, 3P_2_ to synthesis glycerate-3P. The other three orders have complete glycolytic pathway (Figure 1). The result suggests that members of the order myxococcales lack the ability to obtain energy by glycolysis, which is consistent with the fact myxococcales were unable to use glucose as a C source for growth (Sood et al., 2015). In addition, all 17 genomes possessed complete pathways for gluconeogenesis, suggesting they have the potential to convert pyruvate into Glucose-6P as a carbon source and for energy storage (Figure 1). Five genomes *A. dehalogenans* 2CP-1, *V. incomptus* DSM 27710, *Chondromyces crocatus* Cm c5, *Nannocystis exedens* DSM 71 and *Haliangium ochraceum* DSM 14365 lacked key steps for synthesizing histidine (Figure 1). Eleven lacked complete pathways for synthesizing valine, leucine and isoleucine; three lacked complete pathways for synthesizing alanine; four lacked complete pathways for synthesizing arginine; *C. crocatus* Cm c5 have complete pathways for synthesizing tyrosine and phenylalanine and *Polyangium spumosum* DSM 14734 have complete pathways for synthesizing tyrosine (Figure 1). These amino acids are essential for bacterial growth. Bacterial predators were also reported to reduce capacities for synthesizing riboflavin and amino acids (Pasternak et al., 2013). Additionally, known pathway components for *de novo* synthesis of ubiquinone, coenzyme B and thiamin, were not detected in all 17 genomes (Figure 1). The results exhibited that different members of the Myxococcota have multiple metabolic deficiencies.

### The PSSs and the TFF superfamily of the 17 species within the Myxococcota

**Figure 2:**
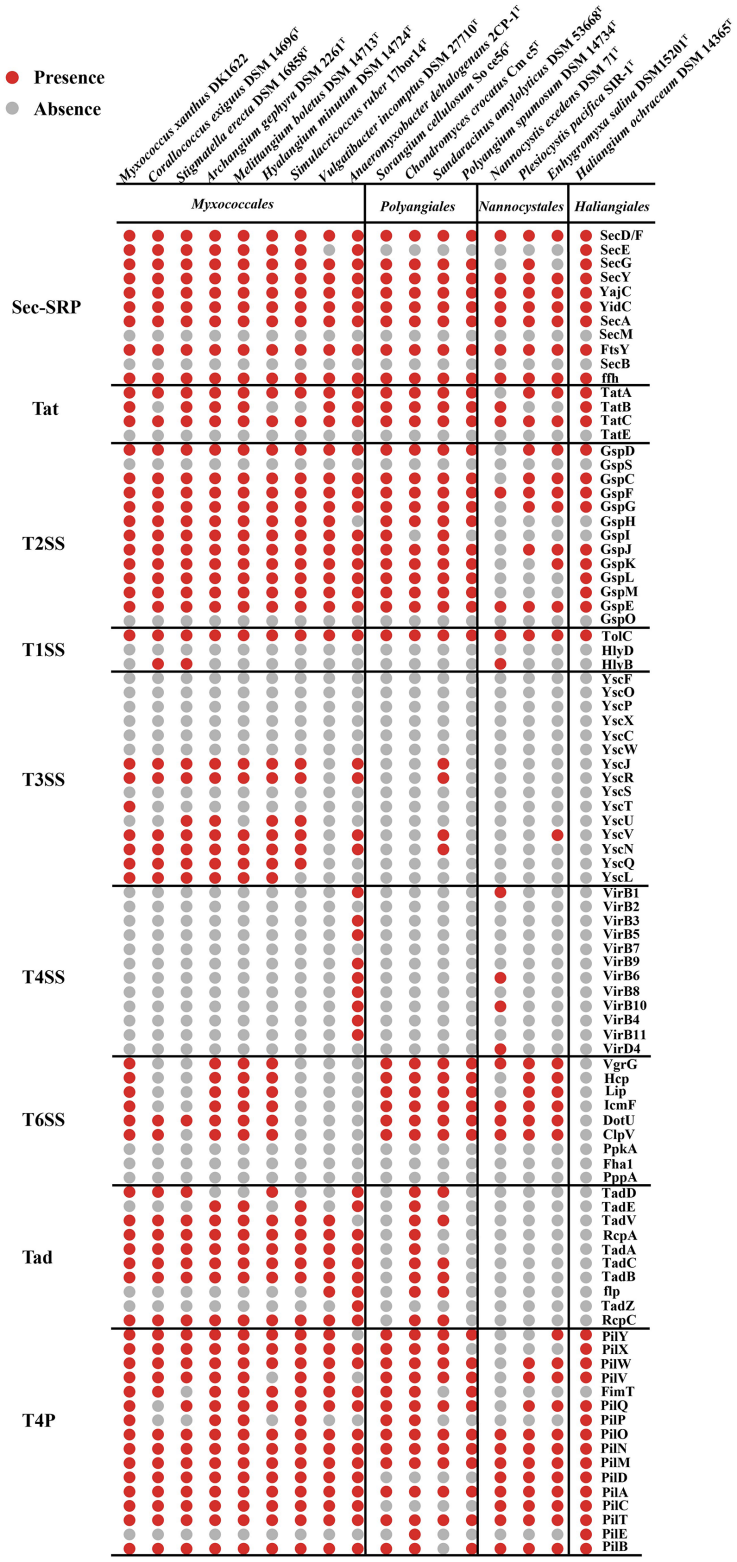
Presence (red dots) or absence (grey dots) of genes within the PSSs and TFF superfamily of 17 species within the Myxococcota. The PSS include two-step secretion systems T2SS, Sec-SRP, Tat, and one-step secretion systems T1SS, T3SS, T4SS and T6SS. The TFF superfamily include T2SS, T4P and Tad pilus.

Bacteria with two membrane bilayers have evolved complex PSSs to export different substances across their cell envelopes (Abby et al., 2016). *Myxococcus xanthus* harbors an intact T2SS, general secretory pathway-signal recognition particle (Sec-SRP) and the twin-arginine translocation (Tat), two degenerate T3SS and an intact T6SS (Konovalova et al., 2010). According to the KEGG annotation and MacSyFinder program (Torres et al., 2014), T2SS, Sec-SRP and Tat system are common within the 17 genomes of the Myxococcota (Figure 2). Many bacterial effectors and toxins depend on the T2SS for secretion (Shutinoski et al., 2010; Sikora et al., 2011). Such substrates are involved in adhesion, biofilm formation, nutrient acquisition, colonization, and invasion (Cianciotto and White, 2017). T1SS, often also referred to as ABC secretion systems or ABC protein exporters, generally consist of two inner membrane proteins (an ATPase, a membrane fusion protein (MFP) and one outer membrane TolC protein) (Kanonenberg et al., 2018), which exist in all 17 genomes (Figure 2). The T3SS, a specialized protein injection complex, acts as a virulence factor in many pathogenic bacteria causing different plant or animal infections (Pourhassan et al., 2021). The T3SS of myxobacteria mainly exist in 9 genomes of the order Myxococcales, which are highly degenerate and none of them seem to encode an intact T3SS (Figure 2). Konovalova et al. also reported the similar results (Konovalova et al., 2010). The T4SS only exist in the family *Anaeromyxobacter* within the order Myxococcales (Figure 2). The T6SS is a contact-dependent weapon that enables direct killing of other cells by translocation of proteinaceous toxins into its competitors (Joshi et al., 2017). *Myxococcus xanthus* use the T6SS-dependent killing heterogeneous populations to eliminate starving or less-fit cells, thus facilitating the attainment of homeostasis within a population and synchronization of behaviors (Troselj et al., 2018). In our analyses, genes encoding T6SS-related proteins mainly exist in the three orders of Myxococcales, Polyangiales and Nannocystales, however, are highly degenerate or absent within some species, such as *Corallococcus exiguus* DSM 14696^T^ and *Stigmatella erecta* DSM 16858^T^ (Figure 2). The bacterial TFF superfamily sinclude the T2SS, the T4P, the Tad pilus and MSH (Denise et al., 2019). The myxobacterial Tad pilus mainly exist in the order Myxococcales and predatory species of the order Polyangiales (Figure 2), indicating they have similar predatory strategies. The T4P are possessed in all four orders, suggesting the Myxococcota have the capacity for T4P-dependent motility (Figure 2).

### Comparative transcriptomics of *Myxococcus xanthus* monoculture compare to coculture with different prey

*Myxococcus xanthus* showed similar predatory behavior towards *E. coli* and *M. luteus* (Supplementary Figures S2A,B). However, the predation for Gram-negative or -positive bacteria are mediated by different bacteriolytic mechanisms (Arend et al., 2020). To further determine the genes involved in predation, we performed comparative transcriptomics when *M. xanthus* was cultured alone (Mx) and cocultured with *E. coli* (MxE) and *M. luteus* (MxM), respectively. More than one hundred and twenty million clean reads were obtained from all 9 subtranscriptomes (Mx1, Mx2, Mx3, MxE1, MxE2, MxE3, MxM1, MxM2 and MxM3) (Supplementary Table S1). The clean data of each subtranscriptome was matched to the reference genome *M. xanthus* DK1622 (CP000113) and most sequences from Mx (66.8–68.2%), MxE (65.7–66.3%) and MxM (68.0–68.2%) were matched (Supplementary Table S1).

**Figure 3:**
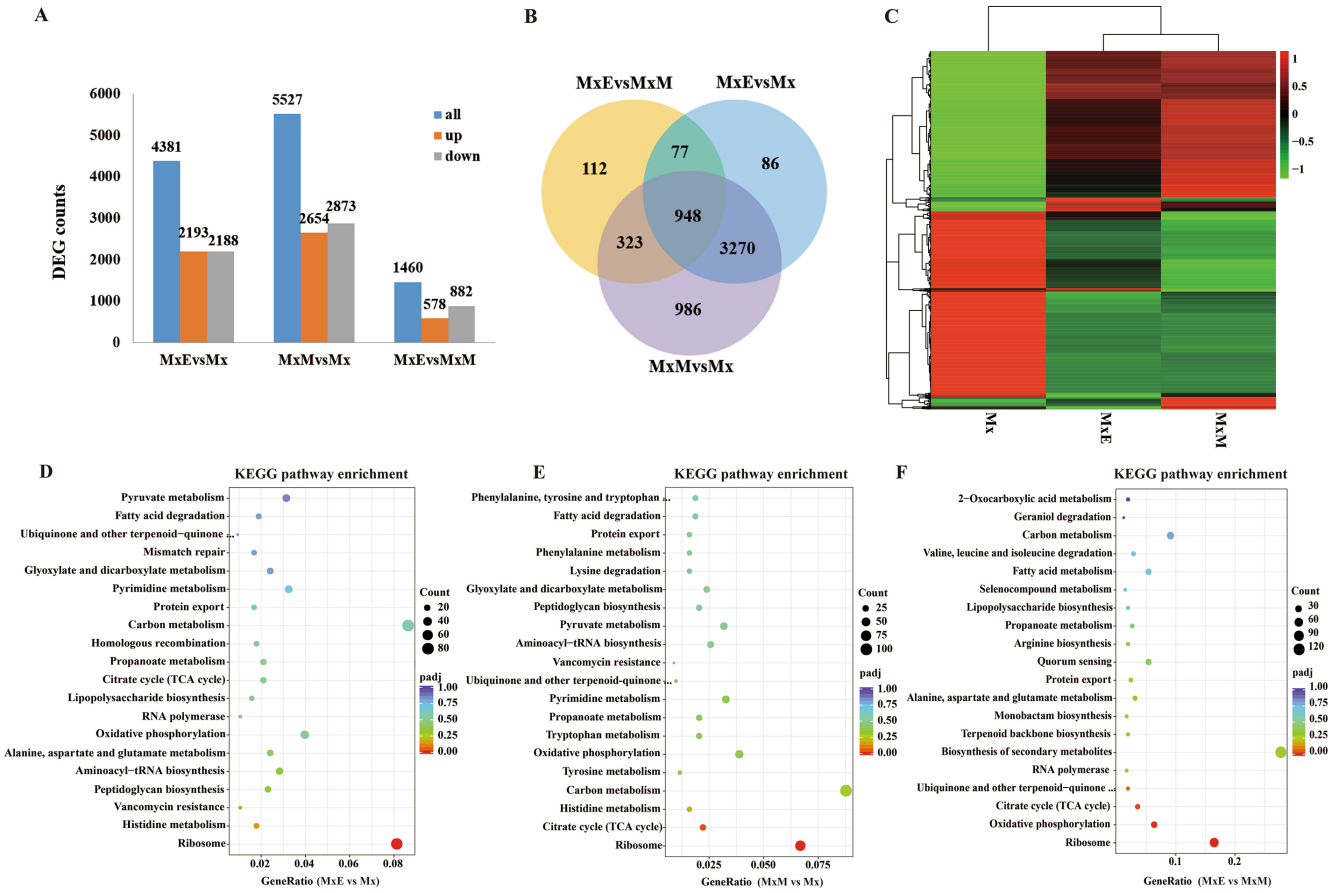
Identification of differentially expressed genes (DEGs). **(A)** The number of DEGs among the three comparisons. **(B)** Venn diagram showing the overlapping DEGs among the three comparisons. **(C)** Heatmap of the DEGs in Mx, MxE and MxM. Color indicates level of relative content of each DEG, from green (low) to red (high). **(D–F)** The KEGG pathway enrichment scatter plots of DEGs in MxE versus Mx **(D)**, MxM versus Mx **(E)** and MxE versus MxM **(F)**. The x-axis represents the ratio of numbers of differential genes annotated in the KEGG pathway to the total number of differential genes, and the y-axis indicates the KEGG pathway. Color scale indicates the expression degree of padj; red, high expression; purple, low expression. The dot sizes represent the gene numbers of enrichment.

In total 4,381 including 2,193 upregulated and 2,188 downregulated DEGs were found between MxE versus Mx. 5,527 including 2,654 upregulated and 2,873 downregulated DEGs were found between MxM versus Mx. Besides, 1,460 including 578 upregulated and 882 downregulated DEGs were found between MxE versus MxM (Figure 3A). We further compared the DEGs by venn diagram analysis and generated 5,802 shared and unique DEGs between Mx and MxE/MxM (Figure 3B). Genes with the same or similar expression patterns are grouped together to identify the unknown functions (Figure 3C). The enrichment analysis of KEGG pathway found that the DEGs were mainly related to ribosomal proteins, carbon metabolism and oxidative phosphorylation among the different treatments (Figures 3D–F). Because *M. xanthus* was cultured alone in WAX agar without any N- and C- sources, these DEGs related to ribosomal proteins, carbon metabolism or oxidative phosphorylation mainly involved myxobacterial growth and development during coculture conditions. In addition, the biosynthesis pathways of secondary metabolites were upregulated or downregulated during predation (Figure 3F), indicating myxobacteria kill Gram-negative or -positive bacteria by secreting different secondary metabolites. As shown in Supplementary Figure S3, the volcano plots and KEGG pathway enrichments of upregulated or downregulated expression showed pairwise comparisons of gene expression among the different treatments.

### Expression analysis of the potential predation DEGs

*Myxococcus xanthus* predation was a multifactorial process, with multiple determinants enhancing predation capacity (Müller et al., 2016; Livingstone et al., 2017). These determinants involved contact-dependent and contact-independent predation process (Müller et al., 2016; Arend et al., 2020; Thiery et al., 2022). We analyzed the potential predatory determinants from these DEGs in three treatments, mainly including the PSSs and TFF superfamily, extracellular enzymes, secondary metabolites, and specifically expressed genes in MxE versus Mx/MxM.

#### The PSSs and the TFF superfamily

**Figure 4:**
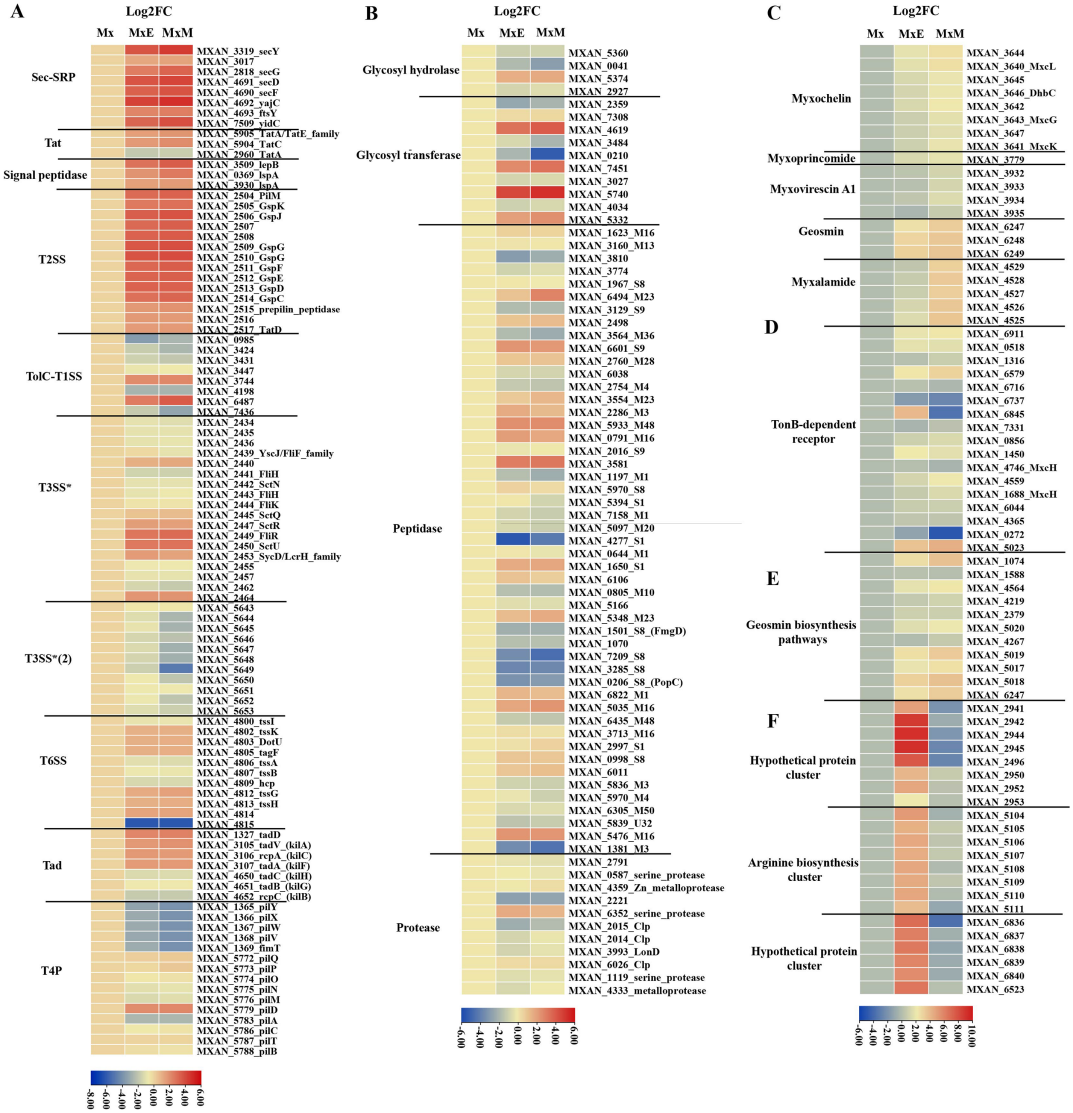
The potential predatory gene expression profiles within Mx, MxE and MxM involved in **(A)** the PSSs and TFF superfamily, **(B)** production of enzymes, **(C)** secondary metabolites biosynthetic clusters, **(D)** TonB-dependent receptors, **(E)** geosmin biosynthesis pathways, **(F)** specifically expressed genes in MxE compared with Mx and MxM. The results were visualized using the “Heatmap” of the TBtools.

*Myxococcus xanthus* cell contact-dependent prey killing involves Tad-like and T3SS* (Seef et al., 2021; Thiery et al., 2022). Two gene clusters *MXAN_2434–2,464* and *MXAN_5643–5,654*, marked as T3SS* and T3SS*(2), encode for T3SS components (Konovalova et al., 2010; Thiery et al., 2022). In our transcriptome data, part of genes encoding T3SS* were upregulated, involving a contributing gene (*MXAN_2445*, *sctQ*), while all genes encoding T3SS*(2) were downregulated (Figure 4A). The results were consistent with reported that T3SS*(2) was not involved in killing of bacterial prey (Thiery et al., 2022). One of the two gene clusters (*MXAN_1327*, *MXAN_3105*-*MXAN_3107*) encoding the Tad-like pilus were significantly upregulated (Figure 4A). Additionally, the expression of all genes encoding signal peptidases and the two-step secretion systems T2SS, Sec-SRP and Tat pathway except *MXAN_2960* were significantly upregulated (Figure 4A), indicating that T2SS probably secrete many hydrolytic enzymes or other substances used to degrade prey biomass during coculture conditions. Conversely, the expression of eight genes encoding TolC-T1SS except *MXAN_3744* and *MXAN_6487* were all downregulated. Furthermore, part of genes encoding the T6SS were upregulated. The T6SS play major roles in the colony boundary formation of *M. xanthus* (Anwar et al., 2019). In addition, most of genes encoding T4P were significantly downregulated (Figure 4A), suggesting that T4P-dependent motility is weakened during predation.

#### The production of enzymes and secondary metabolites-related genes

Another way that myxobacteria kill and digest their prey is by secreting hydrolytic enzymes and antimicrobial substances delivered by OMVs into surrounding environment (Berleman et al., 2014). Genes involved in production of different enzymes (Figure 4B; Muñoz-Dorado et al., 2019) and secondary metabolites (Figure 4C) were examined. Partial glycosyl transferase genes (*MXAN_5740*, *MXAN_4619* and *MXAN_7451*) and peptidase genes (*MXAN_3581*, *MXAN_5933*, *MXAN_6601* and *MXAN_0791*). Only a few genes expression, mainly including glycosyl transferase *MXAN_0210* and peptidase M23 *MXAN_6494*, were significantly differences between MxE and MxM (Figure 4B), indicating these enzymes probably lyse different bacteria. Furthermore, using antiSMASH annotation, all genes encoding five secondary metabolites biosynthesis (myxochelin A/B, myxoprincomide, myxovirescin A1, geosmin and myxalamide) were differentially expressed in cocultures compared to monoculture condition (Figure 4C). The myxoprincomide and myxovirescin A1 have been reported to be involved in predation of *M. xanthus* on *E. coli* and *Bacillus subtilis* (Xiao et al., 2011; Müller et al., 2016; Phillips et al., 2022). Our result also agree with the finding that myxovirescin is overproduction during predator–prey interactions between *M. xanthus* and *E. coli* (Ellis et al., 2019). However, the two clusters are not upregulated in the interaction *M. xanthus* and *Sinorhizobium meliloti*, probably indicate that different antibiotics are overproduced on predating specific prey (Pérez et al., 2022). The gene expression of myxalamide biosynthetic cluster was highly upregulated in MxM versus MxE (Figure 4C), which was consistent with myxalamide being active against Gram-positive bacteria but not Gram-negative bacteria (Charousova et al., 2017). In addition, *M. xanthus* was found to be toxic in the presence/absence of prey and instead regulated feeding when exposed to nutrients from live and dead *E. coli* using mRNA sequencing (Livingstone et al., 2018). The results above provided the potentially opposing observations with our data probably because transcriptome samples were in different stage and experimental treatments. Livingstone et al., did six treatments in liquid states, including *M. xanthus* and living/dead *E. coli* in TPM media, *M. xanthus* alone and live *E. coli* alone in TPM/LBCY media, while we were on solid TPM media using *M. xanthus* alone as control. Additionally, the absence of nutrients in the *M. xanthus* only (no prey) experiments may be the major factor contributing to the observed differences between the studies.

Myxochelin A/B, a representative iron-chelating compound in *M. xanthus*, is a siderophore released under iron-deficient conditions to form a complex with iron. Then, TonB membrane proteins recognize and transport the ferric complex to the cytoplasm, followed by transferring Fe^2+^ from the ferric complex to various enzymes or transcription factors (Cornelis, 2010). During predation, all genes encoding myxochelin A/B biosynthesis cluster and TonB-dependent receptor protein except *MXAN_6737* and *MXAN_0272* were upregulated (Figures 4C,D). The similar results were also found in the interaction *M. xanthus* and *S. meliloti* (Pérez et al., 2022). In addition, the Gram-negative bacterial TonB protein gene *MXAN_6845* was upregulated in MxE and significantly downregulated in MxM versus Mx (Figures 4C,D), indicating *MXAN_6845* play different roles on preying the Gram-negative/positive bacteria. These results above can be speculated that *M. xanthus* produce siderophores for sequestering more iron and prey are in a state of the reduced intracellular iron level, which were similar to the finding that *Streptomyces coelicolor* experienced iron-restricted conditions during coculture with *M. xanthus* (Lee et al., 2020).

Geosmin is an important nontoxic odor component produced by many bacteria, mainly including *Streptomyces*, myxobacteria, and cyanobacteria (Wang et al., 2019). According to our results, all related genes encoding geosmin biosynthesis pathways were upregulated (Figure 4E), indicating geosmin is produced with myxobacterial growth and development during predation. Geosmin had been reported as a warning signal indicating the unpalatability of its producers and reducing predation in a manner that benefit predator and prey (Zaroubi et al., 2022). Therefore, it may be as molecular signaling indicating its producers make a wide range of toxic metabolites. Additionally, geosmin biosynthesis genes were also upregulated with myxobacterial development using transcriptome analysis (Muñoz-Dorado et al., 2019; Pérez et al., 2022). These genes may optimize the scavenging of nutrients or help to genserate nutrients that will trigger germination. In conclusion, these different hydrolytic enzymes and secondary metabolites produced by *M. xanthus* might depend on the prey/culture conditions.

#### Specifically expressed genes in MxE compared with Mx/MxM

Secreted proteins lysed only Gram-positive, but not Gram-negative species (Arend et al., 2020). We further analyzed specifically expressed genes in MxE versus Mx/MxM. Among the potential predation DEGs, three gene clusters including two hypothetical protein gene clusters (*MXAN_2941–2,953* and *MXAN_6,836–6,840*) and one arginine biosynthesis gene cluster (*MXAN_5,104–5,111*) were significantly upregulated in MxE versus Mx, while were slight downregulated in MxM versus Mx (Figure 4F). The arginine biosynthesis cluster (*MXAN_5,104–5,111*) involved in arginine biosynthesis was also upregulated in the interaction *M. xanthus* and *S. meliloti* (Pérez et al., 2022). Hoffmann et al. (2018) proposed that homospermidine lipids, which are bioactive against a panel of microorganisms, are originated from arginine via the putrescine pathway (Hoffmann et al., 2018). Additionally, the two hypothetical gene clusters above played unknown functions in the interaction of *M. xanthus* and *E. coli*.

In order to confirm the accuracy of transcriptome data, we selected 12 DEGs from myxochelin A/B, myxalamide and geosmin biosynthesis clusters for qRT-PCR analysis. The expression patterns of these genes obtained from qRT-PCR data have good agreement with transcriptome sequencing data (Supplementary Figure S4). All gene-specific primers for qRT-PCR are listed in Supplementary Table S2.

### The genes distribution of tad-like pilus and five secondary metabolites above in different predators

**Figure 5:**
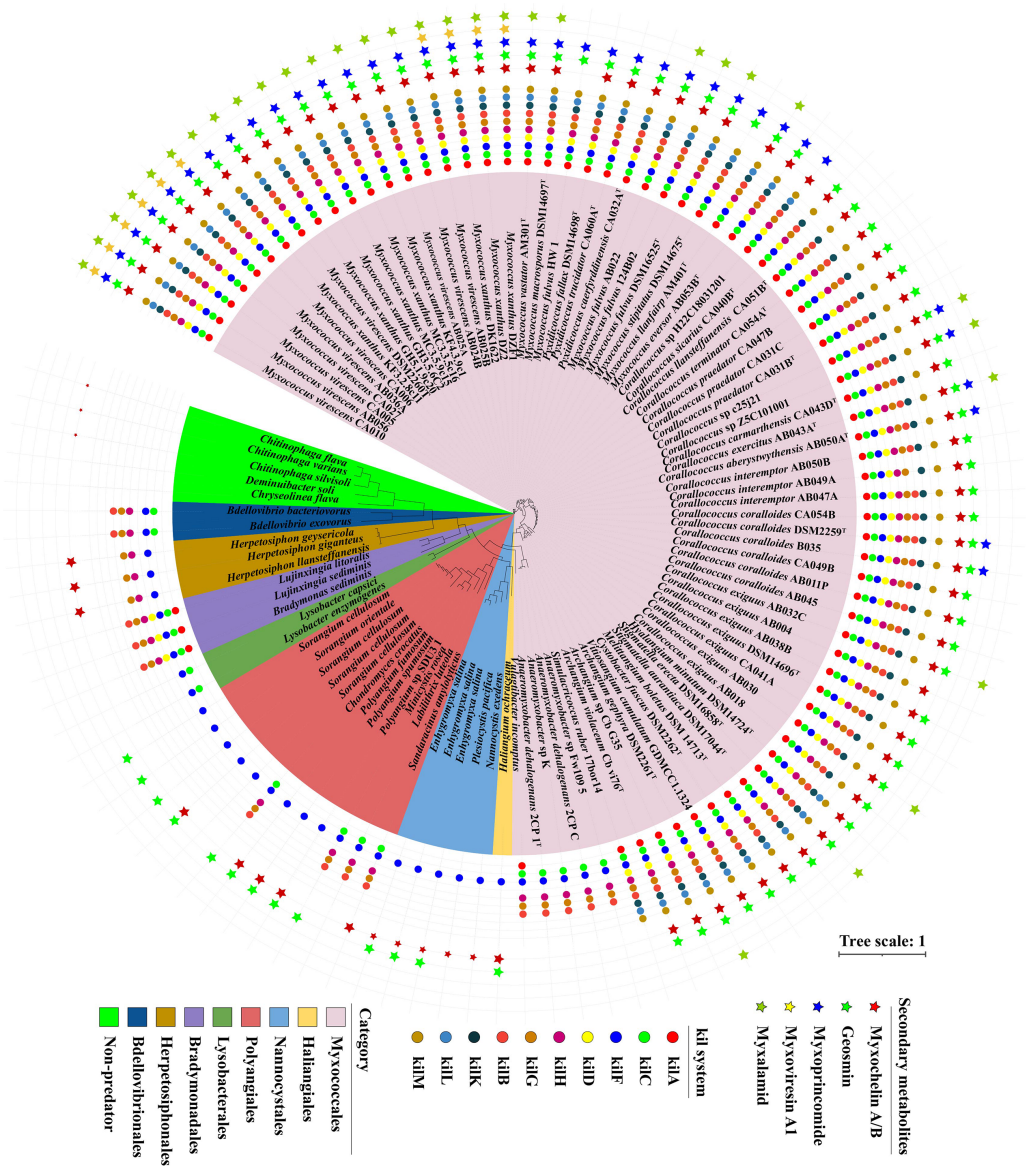
The genes distribution related to Tad (kil) pilus and five secondary metabolites in different predators. A maximum-likelihood (ML) phylogenetic tree was reconstructed based on 92 up-to-date bacterial core genes sequences of predatory and non-predatory bacteria. Predatory bacteria included four orders (Myxococcales, Haliangiales, Nannocystales and Polyangiales) within the phylum Myxococcota, and other four orders (Lysobacterales, Bradymonadales, Herpetosiphonales and Bdellovibrionales). Non-predatory bacteria included two orders (Cytophagales and Chitinophagales). Different groups are indicated and marked using different colors. Circle indicated 10 different genes belonging to Tad (kil) pilus. Star indicated 5 different secondary metabolites annotated by antiSMASH.

Based on our transcriptomic results and the contact-dependent prey killing role of the Tad (kil)-like pilus (Seef et al., 2021), we selected 107 genomes including 92 myxobacteria, 10 other predatory bacteria and 5 non-predators to explore the presence of Tad-like pilus and five secondary metabolites above. General features of these genomes were described in Supplementary Table S3. Homologue genes encoding Tad-like pilus were present in the order of Myxococcales and four genera (*Chondromyces*, *Minicystis*, *Labilithrix* and *Sandaracinus*) of the order Polyangiales (Figure 5), indicating these species have similar predatory strategies. However, homologue genes of Tad-like pilus were not present in the two genera *Sorangium* and *Polyangium* of the order Polyangiales, and the two orders Haliangiales and Nannocystales (Figure 5), indicating these species may have different predatory strategies (or none whatsoever) (Reichenbach et al., 2006). Moreover, the Tad-like genes were also present in the Bdellovibrionales, Bradymonadales and Herpetosiphonales (Figure 5). The result was consistent with reported by Seef et al. (2021) and Thiery et al. (2022). In addition, three antibiotics myxoprincomide, myxovirescin A1 and myxalamide were only present in the order Myxococcales, mainly the genus of *Myxococcus*, while myxochelin A/B and geosmin were present in all four orders of the phylum Myxococcota with the exception of several species (Figure 5). Moreover, myxochelin A/B was also present in the genus of *Herpetosiphon* (Figure 5). The results indicated that antibiotics probably play accessory roles during predation. Moreover, other obligate or facultative predators share similar predatory strategies with myxobacteria.

## Conclusion

In this study, we used comparative genomics and transcriptomics to show the metabolic potentials and the DEG profiles of *M. xanthus* monoculture compared to coculture with *E. coli* (MxE) and *M. luteus* (MxM) prey. We further explored the myxobacterial and other bacterial genomes for the presence of potential predatory effectors. The results indicated that different members of the Myxococcota had conspicuous metabolic deficiencies, and encoded various PSSs, of which intact T2SS were detected (Figures 1, 2). Additionally, *M. xanthus* overexpressed the potential predation DEGs, including those encoding T2SS, T3SS*, Tad-like pilus, secondary metabolites (myxochelin A/B, myxoprincomide, myxovirescin A1, geosmin and myxalamide), glycosyl transferases and peptidase (*MXAN_6601*, *MXAN_5933* and *MXAN_0791*, etc.) during predation (Figure 4). Moreover, two hypothetical protein clusters and an arginine biosynthesis gene cluster (*MXAN_2941–2,953*, *6,836–6,840* and *5,104–5,111*) were significantly upregulated in MxE versus Mx/MxM. Furthermore, the homologue genes of Tad-like pilus and 5 secondary metabolites above were distributed in the different obligate or facultative predators (Figure 5).

**Figure 6:**
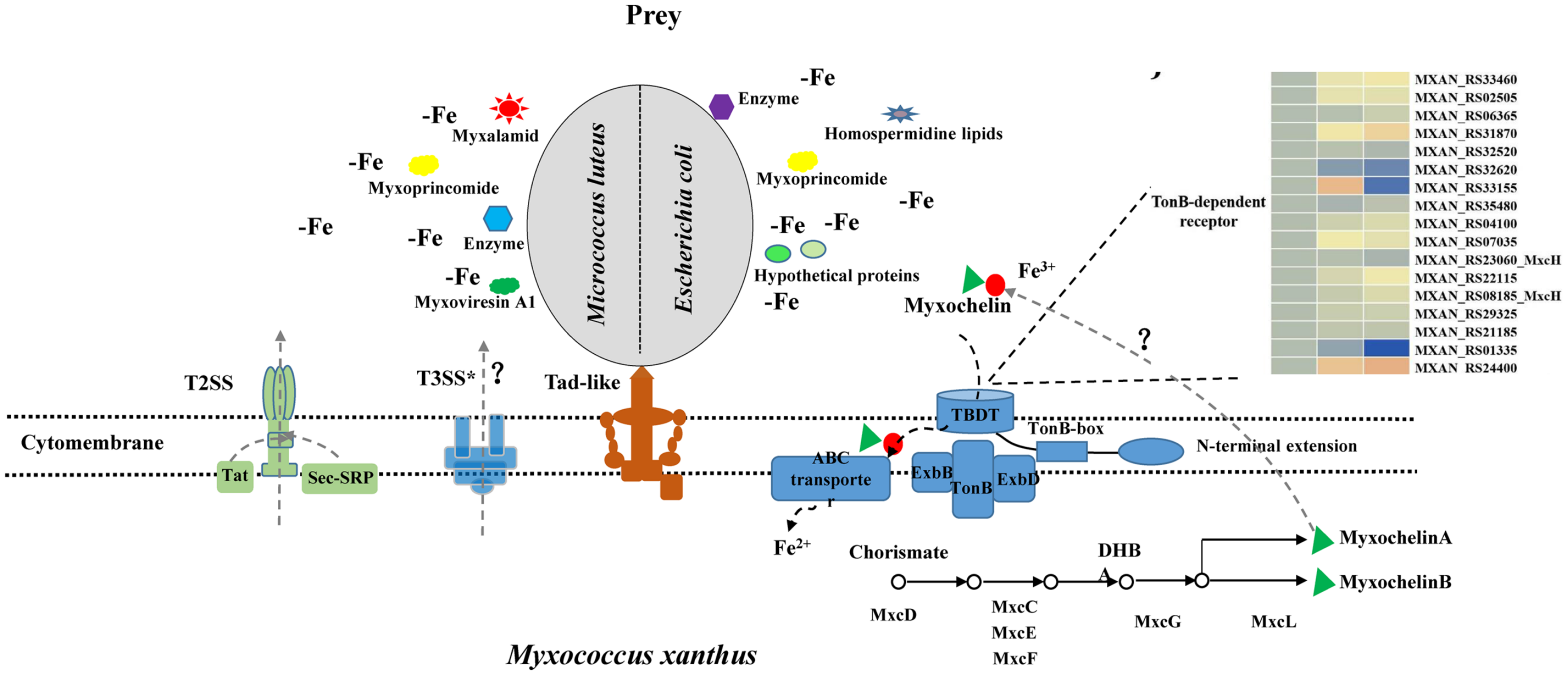
Model depicting how *M. xanthus* predate *M. luteus* and *E. coli*. *M. xanthus* kill prey via the Tad (kil) pilus during contact and then lyse prey through T3SS*. In addition, two-steps secretion systems T2SS, Sec-SRP and Tat pathways probably secrete different lyases, antibiotics or hypothetical proteins into extracellular environment when encountering Gram-positive or -negative bacteria. These substances increase myxobacterial predation efficiency. Meanwhile, myxobacteria produce myxochelin A/B for sequestering more iron by competition sensing, and prey are in a state of the reduced intracellular iron level. Geosmin are produced with myxobacterial growth and development during predation.

According to our results and previous work in the literature, we provided a model of *M. luteus* and *E. coli* killing and lysis by *M. xanthus* (Figure 6). *Myxococcus xanthus* kill prey by Tad-like pilus first and then lyse prey cells through T3SS* (Seef et al., 2021; Thiery et al., 2022). In addition, two-steps secretion systems T2SS, Sec-SRP and Tat pathways probably secrete some lyases, antibiotics or hypothetical proteins into extracellular environments. *M. xanthus* might secrete various predatory effectors such as myxalamide, myxoprincomide, myxoviresin A1 and protease M23 for *M. luteus*, or myxoprincomide, hypothetical proteins and homospermidine lipids for *E. coli* during preying on different prey. These different secretory systems determine whether myxobacteria are predator or not, while different secreta affect myxobacterial predation efficiency. Meanwhile, myxobacteria sequesters more iron for the growth by activating the production of myxochelin A/B and the myxochelin-mediated iron acquisition system, while prey bacteria nearby are in a state of the reduced intracellular iron level. Materials released from the besieged prey cells are then absorbed by the predatory myxobacteria as nutritions. Furthermore, geosmin is produced with myxobacterial growth and development during predation. The complex predation strategies confer an advantage to myxobacteria that enables them to survive in various environments and degrade organic matter or prey bacteria for their growth.

## Data availability statement

The datasets presented in this study can be found in online repositories. The names of the repository/repositories and accession number(s) can be found in the article/Supplementary material.

## Author contributions

CW: conceptualization, investigation, visualization, writing–original draft, and funding acquisition. YX and YL: investigation, methodology, and validation. YW: investigation, methodology, and funding acquisition. QY: writing – review and editing. HZ: conceptualization, writing – review and editing, and supervision. All authors contributed to the article and approved the submitted version.

## Funding

This work was jointly supported by the Natural Science Foundation of China (32001115), the GDAS’ Project of Science and Technology Development (2020GDASYL-20200103032) and Initial Funding for Doctoral Research of Huizhou University (2022JB087), and the Science and Technology Program of Shaoguan (220805106270728).

## Conflict of interest

The authors declare that the research was conducted in the absence of any commercial or financial relationships that could be construed as a potential conflict of interest.

## Publisher’s note

All claims expressed in this article are solely those of the authors and do not necessarily represent those of their affiliated organizations, or those of the publisher, the editors and the reviewers. Any product that may be evaluated in this article, or claim that may be made by its manufacturer, is not guaranteed or endorsed by the publisher.

## Footnotes

^1^ http://www.bacterio.net/myxococcota.html

^2^ https://www.ncbi.nlm.nih.gov/

^3^ https://www.kegg.jp/blastkoala/

^4^ http://macsyfinder.readthedocs.io/en/latest/index.html
