# Supplementary material for: Comparative genomics and transcriptomics insight into myxobacterial metabolism potentials and multiple predatory strategies
Source: Front Microbiol. 2023 May 5;14:1146523. doi: 10.3389/fmicb.2023.1146523 (PMC10196010; doi:10.3389/fmicb.2023.1146523)

**SUPPLEMENTARY FIGURES**

**Comparative genomics and transcriptomics insights into myxobacterial metabolism potentials and multiple predatory strategies**

**Chunling Wang^1, 2^, Yi Xiao^2^, Yong Wang^2^, Yumin Liu^2^, Qing Yao^3^ and Honghui Zhu^2,^ ***

^1^College of Life Science, Huizhou University, Huizhou 516007, Guangdong, P. R. China

^2^Key Laboratory of Agricultural Microbiomics and Precision Application (MARA), Guangdong Provincial Key Laboratory of Microbial Culture Collection and Application, Key Laboratory of Agricultural Microbiome (MARA), State Key Laboratory of Applied Microbiology Southern China, Institute of Microbiology, Guangdong Academy of Sciences, Guangzhou 510070, Guangdong, P. R. China

^3^College of Horticulture, South China Agricultural University, Guangdong Province Key Laboratory of Microbial Signals and Disease Control, Guangzhou 510642, Guangdong, P. R. China

**Running title: Myxobacterial Predatory Strategies**

**Corresponding author:**

**Honghui Zhu; +86-020-87685669; zhuhh_gdim@163.com**

**Figure S1** The genomic general features of 17 species of four orders (Myxococcales, Polyangiales, Nannocystales and Haliangiales) within the Myxococcota. A phylogenomic tree was reconstructed by using 16S rRNA gene sequences. Bar, 0.10 substitutions per nucleotide position. Tree in red background indicate the order Myxococcales, tree in blue background indicate the order Polyangiales, tree in violet background indicate the order Nannocystales, tree in aurantius background indicate the order Haliangiales.

**Figure S2** (A) Predation behavior of *M. xanthus* against *E. coli* (MxE) and *M. luteus* (MxM). *M. xanthus* was spotted on (a-b) and next to (c-d) prey bacteria. *Mx*: *M. xanthus*, *Ec*: *E. coli* and *Ml*: *M. luteus*. Scale bar 1 mm. (B) Light micrographs of MxE and MxM. Cells were stained by crystal violet at 4, 9 and 12 h, respectively.


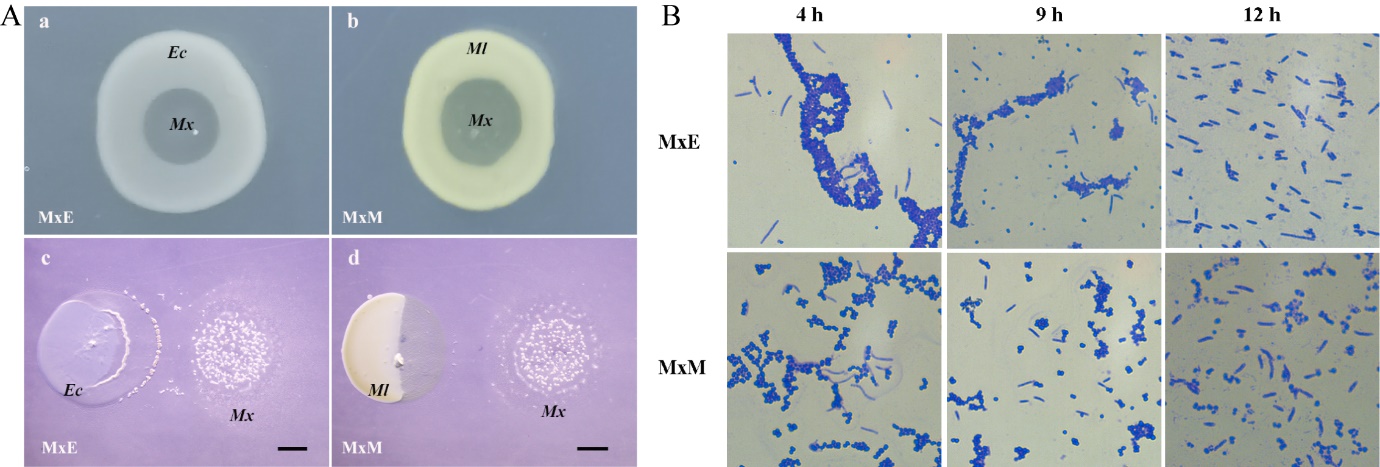


**Figure S3** The potential predation DEG profiles of *M. xanthus* under monoculture and coculture with prey. (A-C) The volcano plots show the estimated fold changes (x-axis) versus the minus log10 of the adjusted p-values (y-axis) from DEGs analysis. (D-I) The KEGG pathway enrichment scatter plots indicate the ratio of numbers of differential genes annotated in the KEGG pathway to the total number of differential genes (x-axis) versus the KEGG pathway (y-axis). Color scale indicates the expression degree of padj; red, high expression; purple, low expression. The dot sizes represent the gene numbers of enrichment. (A, D and G), indicate in MxE vs Mx. (B, E and H), indicate in MxM vs Mx. (C, F and I), indicate in MxE vs MxM.


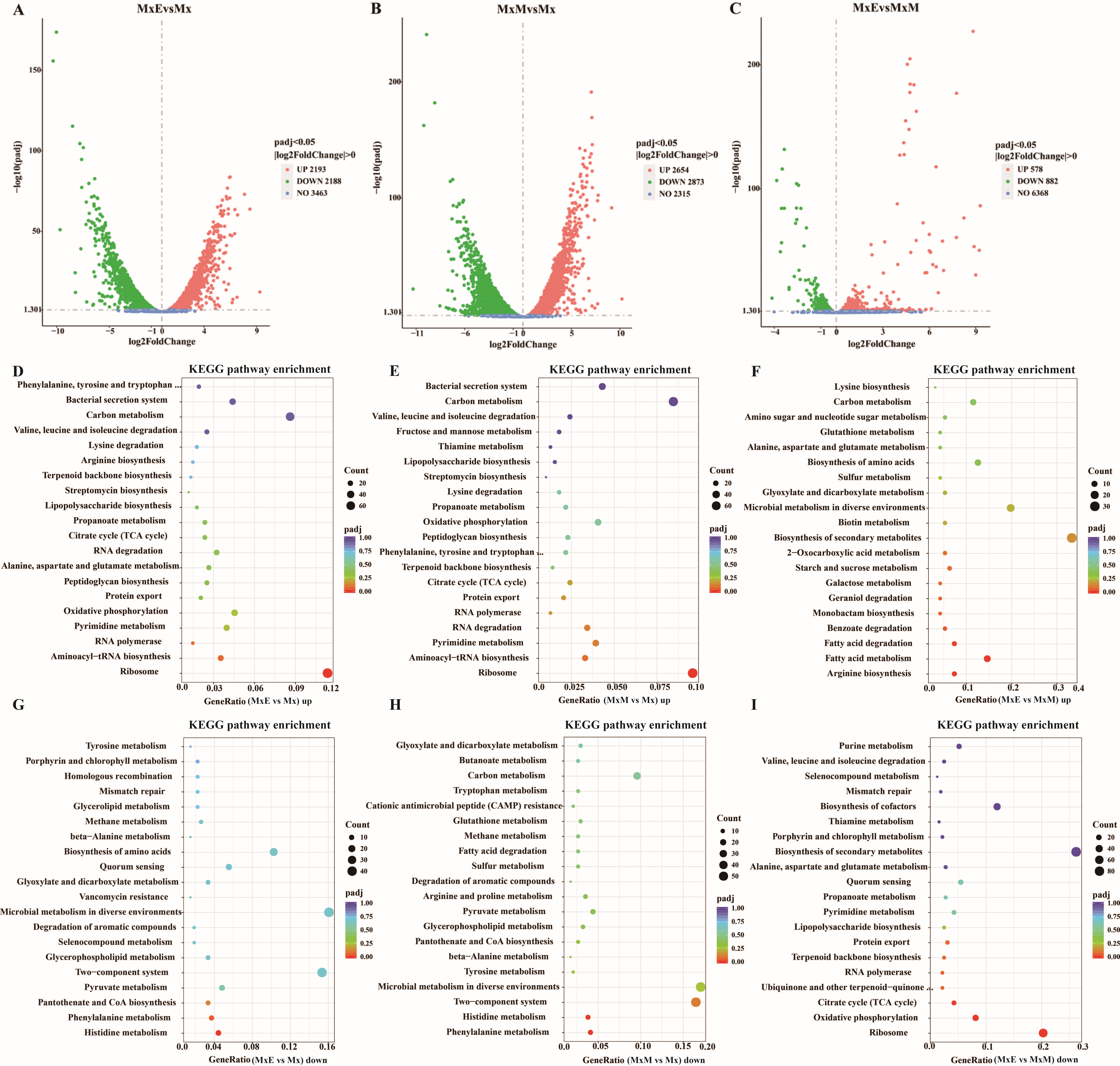


**Figure S4.** (A) Gel electrophoresis image of 12 gene primers for qRT-PCR. (B) The relative expression level of 12 genes related to secondary metabolite clusters under Mx, MxE and MxM conditions. The relative expression level of these genes at Mx condition was employed as internal control.


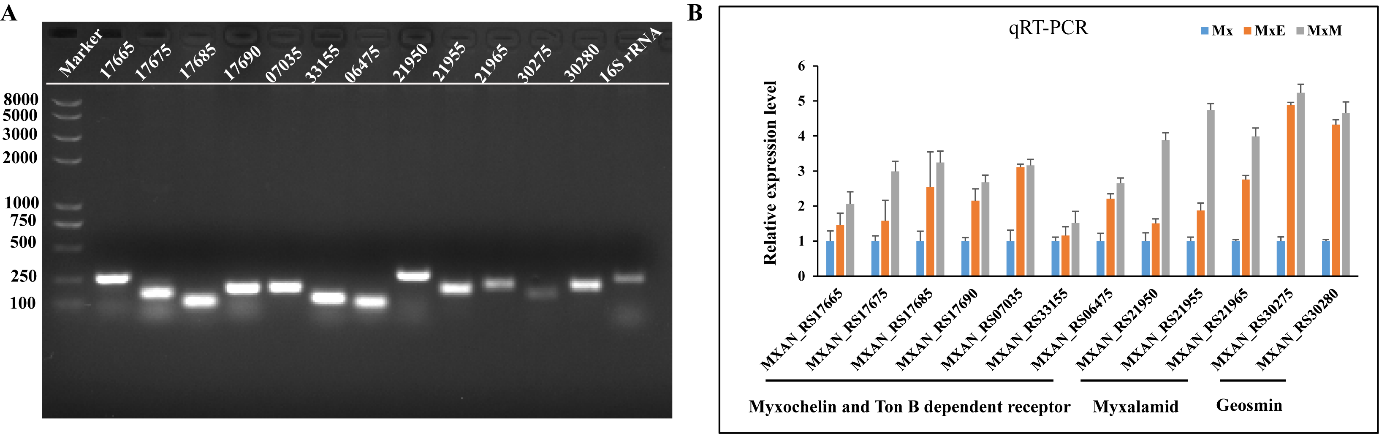

Supplement: Supplementary file 2 [file Data_Sheet_1.docx]
